# The cost-effectiveness of whole genome sequencing in neurodevelopmental disorders

**DOI:** 10.1038/s41598-023-33787-8

**Published:** 2023-04-27

**Authors:** Hannes Runheim, Maria Pettersson, Anna Hammarsjö, Ann Nordgren, Martin Henriksson, Anna Lindstrand, Lars-Åke Levin, Maria Johansson Soller

**Affiliations:** 1 Department of Health, Medicine and Caring Sciences, Linköping University, Linköping, Sweden; 2 Department of Clinical Genetics, Karolinska University Hospital, Stockholm, Sweden; 3 Department of Molecular Medicine and Surgery and Center for Molecular Medicine, Karolinska Institutet, Stockholm, Sweden; 4 Genomic Medicine Center Karolinska, Karolinska University Hospital, Stockholm, Sweden

**Keywords:** Genomics, Neurodevelopmental disorders, Genetics, DNA sequencing, Next-generation sequencing, Genetic testing, Health care economics

## Abstract

Whole genome sequencing (WGS) has the potential to be a comprehensive genetic test, especially relevant for individuals with neurodevelopmental disorders, syndromes and congenital malformations. However, the cost consequences of using whole genome sequencing as a first-line genetic test for these individuals are not well understood. The study objective was to compare the healthcare costs and diagnostic yield when WGS is performed as the first-line test instead of chromosomal microarray analysis (CMA). Two cohorts were analyzed retrospectively using register data, cohort CMA (418 patients referred for CMA at the department of Clinical Genetics, Karolinska University Hospital, during 2015) and cohort WGS (89 patients included in a WGS-first prospective study in 2017). The analysis compared healthcare consumption over a 2-year period after referral for genetic testing, the diagnostic yield over a 2- and 3-year period after referral was also compiled. The mean healthcare cost per patient in cohort WGS was $2,339 lower compared to cohort CMA ($ − 2339, 95% CI − 12,238–7561; P = 0.64) including higher costs for genetic investigations ($1065, 95% CI 834–1295; P < 0.001) and lower costs for outpatient care ($ − 2330, 95% CI − 3992 to (− 669); P = 0.006). The diagnostic yield was 23% higher for cohort WGS (cohort CMA 20.1%, cohort WGS 24.7%) (0.046, 95% CI − 0.053–0.145; P = 0.36). WGS as a first-line diagnostic test for individuals with neurodevelopmental disorders is associated with statistically non-significant lower costs and higher diagnostic yield compared with CMA. This indicates that prioritizing WGS over CMA in health care decision making will yield positive expected outcomes as well as showing a need for further research.

## Introduction

Neurodevelopmental disorders (NDDs), including intellectual disability (ID), autism spectrum disorders, attention-deficit hyperactivity disorder, attention-deficit disorder, seizures and speech development disorders, have been estimated to affect around 15% of children in the United States^1,2^. The ID subgroup (prevalence ~ 1%)^3^ has intellectual function as measured by an Intelligence Quotient (IQ) test below 70 as well as limitations in adaptive behavior^4^. A large proportion of NDD cases have an underlying genetic cause, especially those with moderate and severe ID (> 70% genetic cause for those with IQ < 50)^5–8^. Rare variants in over 1000 genes may cause ID^9^ as well as deletions and duplications and repeat expansions^7^. Other even more rare molecular events include balanced structural chromosomal rearrangements (such as reciprocal translocations and inversions) and complex genomic rearrangements. For other NDDs as well as the mild ID cases the underlying etiology is more complex but genetic causes are still important and such causes include a multitude of genetic variants.

The importance of a genetic diagnosis is twofold; (i) for the individual affected by NDD, a molecular diagnosis enables personalized treatment and follow up; (ii) for the family members it provides access to carrier testing and family planning. Since co-morbidities are common^10^ a prompt and correct genetic diagnosis generated by applying whole exome sequencing early in the diagnostic trajectory has been estimated to be cost-effective, an effect of a reduction in the overall number of genetic tests needed^11,12^. Furthermore, the knowledge of a specific diagnosis reduces anxiety and stress for the family^13^, which in turn also could potentially reduce the amount of healthcare visits. However, since the available data from such analyses are based on exome sequencing, which mainly detects single nucleotide variants (SNVs) and insertions/deletions (INDELs) in single genes, it is not a method that could replace chromosomal microarray (CMA) or short tandem repeat expansion (STR) screenings.

Whole genome sequencing (WGS) is an umbrella term for a wide range of sequencing technologies that are used to sequence large parts of the genome, including the non-coding parts of genes and intergenic regions. The WGS method used worldwide is what is generally referred to as “next-generation sequencing”, a short-read massive parallel sequencing (MPS) technology that sequence the genome, which is subsequently mapped back to the reference genome. The bioinformatic tools for analysis and detection of genetic variation is rapidly developing and a combination of tools allows for detection of SNVs/INDELS^14^, structural variants (SVs) including copy number variants (CNVs, deletions and duplications) and balanced rearrangements (translocations, inversions)^15–17^, STRs^18^, and uniparental disomy/loss of heterozygosity regions (isodisomy for singleton cases as well as heterodisomy for trios)^7,8^ in a single analysis, which has revolutionized genetic diagnostics.

The availability for WGS analysis is rapidly improving worldwide, and the cost per analysis including the bioinformatics has dramatically dropped the last 10 years. With the increasingly sophisticated tools for genetic variation detection, a single WGS analysis can today replace many traditional genetic analysis tools, including Sanger sequencing, CMA and STR screening (i.e. *FMR1*, Fragile X syndrome and several spinocerebellar ataxia disorders). Currently, in Stockholm County (Sweden) including the department of Clinical Genetics, Karolinska University Hospital, the first-line test for individuals with NDDs is still commonly CMA (and *FMR1* screening when CMA is negative) but WGS is requested more and more often. Our recent work shows that by using WGS as the first line test in such cases higher diagnostic yields and lower costs per diagnosed individual are achieved compared to CMA as the first line test^19^.

Health economic studies with implementation of WGS as a diagnostic tool are still sparse but some studies are beginning to emerge. In a review article from 2018, investigating the cost-effectiveness of sequencing with WGS or exome sequencing, 36 articles (of which 12 were related to WGS) were identified^20^. Although most of them indicated that sequencing was cost-effective the studies were often based on a small number of individuals or difficult-to-diagnose patient groups, focused on certain economic aspects or presented results that may rapidly become outdated. More recently, the American College of Medical Genetics have suggested in their clinical guidelines that WGS is a suitable first or second tier test for patients with ID^21^. Finally, recent research also shows an increased diagnostic yield in monogenic disorders after WGS and consequently both lower costs and a lower number of tests needed^22^.

This study aimed to analyze the cost-effectiveness of WGS in comparison to CMA as the first-line genetic diagnostic test in an unselected group of individuals who were referred to clinical genetics at Karolinska University hospital for CMA analysis.

## Materials and methods

### Study design

This study was designed as a retrospective observational study that compared historical data on healthcare costs, diagnostic yield and type of genetic tests between two cohorts of patients referred to the department of Clinical Genetics at the Karolinska University Hospital. One cohort (cohort CMA) represented standard care, with CMA as the first-line diagnostic test, and the other cohort (cohort WGS) represented testing with WGS as the first-line diagnostic test. Costs were collected under a period of 2 years starting with the referral date. Diagnostic yield and genetic test information were collected over a period of up to 3 years from referral.

### Study subjects

The Swedish health care system is primarily publicly financed and administered and is available to all residents. Clinical Genetics (Karolinska University Hospital) provides diagnostic genetic testing and genetic counselling for patients from the greater Stockholm region which has approximately 2.4 million permanent residents (representing 23% of the Swedish population). Approximately 500–600 individuals with NDDs are referred for genetic testing each year. In recent years, the genetic testing strategy used for individuals with NDD has changed and today singleton WGS is more and more often requested instead of CMA as the first line diagnostic test.

The inclusion criteria for both cohorts in this study, were patients referred to our center for CMA. We included all referrals during 2015 (cohort CMA) and the first 100 in 2017 (cohort WGS). Individuals that were referred from outside Stockholm County or were born before the year 2000 were excluded. These exclusion criteria were set to ensure participance in the VAL-databases (see below), as well as to ensure anonymity. After exclusion, cohort CMA included 418 out of 529 individuals referred for CMA during 2015 and cohort WGS included 89 of the first 100 individuals referred for CMA in the first quarter of 2017. Those first 100 individuals were in parallel with CMA investigated with WGS as part of a clinical development project^7^.

### CMA

The CMA used was a 4 × 180 K custom oligonucleotide microarray with an even whole-genome coverage and median probe spacing of approximately 18 kb. Deletions and duplications were called with three consecutive aberrant probes, hence giving the resolution of the CMA analysis approximately 50 kb.

### WGS

In brief, we used a short-read PCR-free paired-end protocol (Illumina TruSeq DNA PCR-free) and sequenced on the NovaSeq 6000 platform (Illumina, San Diego, California, USA) with 30× median coverage. Detailed description of sequencing and data analysis was reported previously^7,8^ and included bioinformatic analysis of SNVs/INDELs, SVs (including both unbalanced (deletions/duplications) as well as balanced (translocations/inversions)) and STR expansions in genes with known STR expansion disease mechanism.

Importantly, WGS was performed as a complementary analysis to standard of care. Both sequencing and data analysis was done in the research setting and pathogenic variants detected were validated and reported clinically throughout the project with the final variants reported at the end of the study (1.5 years later)^7^.

### Data sources

Health economic data and genetic results regarding the two cohorts were retrieved from two sources. The first data set consisted of an anonymized excerpt from the VAL databases. The VAL databases are a set of databases that aims to create an overview of an individual’s total healthcare consumption over time within Stockholm County^23,24^. The data set consisted of information regarding both outpatient and inpatient health care and included, among other things, age, sex, dates of health care visits and their costs. The inpatient care costs included, among other things, costs due to hospital stays, treatments, surgeries, and in-hospital drugs. The outpatient care costs included costs due to health care visits of various types but not prescription drugs. A second anonymized data set was extracted from the department of Clinical Genetics management system StarLims (Abbott Informatics). It complemented the VAL excerpt with additional costs and information regarding the individual’s genetic investigation generated at the department. The data set contained information about the types of genetic tests performed, dates, costs as well as information regarding molecular genetic diagnoses.

### Statistics and analysis

Descriptive statistics of costs and diagnostic yield of cohort CMA and cohort WGS, respectively, were reported. Central tendencies were described as means and differences in means were tested with t-tests (not assuming equal variances). The required level to attain statistical significance was set to P = 0.05. Calculations were performed in Microsoft Excel 365:2201 (Microsoft Corporation, Redmond, Washington DC, USA) and SPSS 28 (IBM Corp. Released 2021. IBM SPSS Statistics for Windows, Version 28.0. Armonk, NY: IBM Corp).

The costs were calculated from each individual's referral date for CMA and 2 years onwards. They were adjusted for inflation (target year 2020) based on the County Council Price Index (LPI)^25^ and converted from SEK to USD with the average exchange rate of 2020 (9.2037 SEK = 1 USD)^26^.

Since cohort WGS received standard of care with CMA in parallel with the WGS-testing strategy, the CMA test and, where applicable, the subsequent genetic tests that presently can be replaced by a WGS test were excluded from the analysis. The assessment of which tests to exclude was made in collaboration with clinical laboratory geneticists at the department of Clinical Genetics, Karolinska University Hospital. WGS was estimated to replace the CMA, FMR1-screening (Fragile X) and exome tests as well as a few instances of other, rarely occurring, panel/sequencing tests. The diagnoses identified with WGS were in some cases verified with additional tests as the method was still under evaluation. The need for these extra verifications is currently estimated to have decreased by 85% and the amount of verification tests were therefore lowered by said proportion.

To ensure that seasonal variations in costs did not affect the cost analysis, the follow-up period for the cohorts was set to full years. A balance check was performed for cohort CMA and WGS regarding mean age and proportion of women. Also, since cohort CMA consisted of referrals during 2015 and cohort WGS of referrals from 2017, we calculated the cost differences between the cohorts 1 year prior to respective cohort referral year (2014 and 2016), hypothesizing that these would be similar if the cohorts were comparable. The effect of age and sex on total costs was analyzed using a multiple linear regression model.

The diagnostic yield in respective cohort was calculated for two time points, (i) after 2 years from referral and (ii) after 3 years from referral. It was possible to add an additional year of follow up since the data regarding the respective cohort’s diagnostic yield was further extended in time than the cost data.

The cost-effectiveness analysis (CEA) compared the mean differences in cost and diagnostic yield when using WGS instead of CMA as the first-line genetic diagnostic test, measuring from the date of the referral and with a time horizon set to 2 years. Costs and diagnostic yield were calculated from the perspective of the health care system and were not discounted, due to the short timeframe. The incremental cost-effectiveness ratio (ICER) describes the additional cost required to produce an additional genetic diagnosis. If the use of the WGS is shown to be both less costly and provides a larger diagnostic yield, the use of WGS is dominant and no ICER is calculated.

A sensitivity analysis was performed using assumptions of future lower WGS-verification testing and WGS-testing cost. The WGS verification test costs were set to zero and WGS-testing cost to 50%. Lastly, we calculated how much lower the WGS testing costs would have to be in cohort WGS, in order for total genetic costs to be equal in the cohorts (given verification testing costs set to zero).

### Ethics approval and consent to participate

Ethics approval was given by the Regional Ethical Review Board in Stockholm, Sweden (ethics permit numbers KS 2012/222-31/3 and 2012/2106-31/4). This ethics permit allows for use of clinical samples for analysis of scientific importance as part of clinical development. Included subjects were part of clinical cohorts investigated at our center. IRB approval for informed consent to participate in the study was waived by the by the Regional Ethical Review Board in Stockholm, Sweden. The research conformed to the principles of the Helsinki Declaration.

## Results

**Figure 1:**
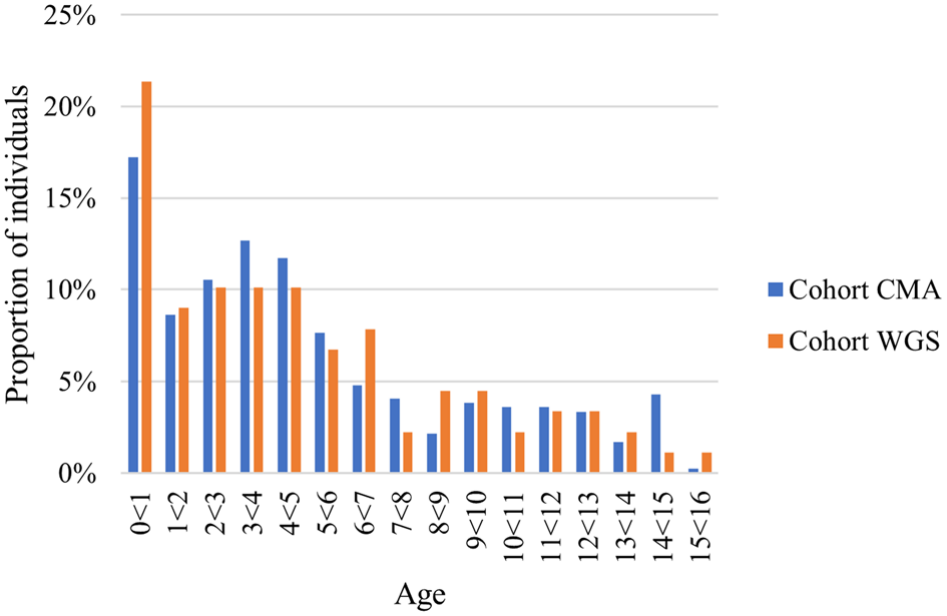
Age distribution upon referral.

A summary of the cohort characteristics is given in Table 1 and the age distribution in Fig. 1. There were no significant differences regarding mean age (P = 0.54) and the proportion of women (P = 0.53) between the two cohorts.

**Table 1:** Cohort characteristics at time of referral.

Cohort	WGS (n = 89)	CMA (n = 418)
Sex (female)	37% (n = 33)	33% (n = 140)
Age (mean years; min/max/median)	4.8; 0.0/15.3/3.8	5.1; 0.0/15.5/4.0
Main reason for referral^a^		
Neurodevelopmental disorder	81% (n = 72)	82% (n = 342)
Affected newborn (< 3 months)	13% (n = 12)	10% (n = 40)
Other^b^	6% (n = 5)	11% (n = 48)

^a^Twelve newborns in cohort CMA showed developmental delay that was obvious as early as under three months of age, hence twelve patients are included in both Neurodevelopmental disorder group and Affected newborn (< 3 months) group in Table 1, hence the percentages will not add up.

^b^Epilepsy, congenital malformations or suspected neuromuscular disease.

### Costs

**Figure 2:**
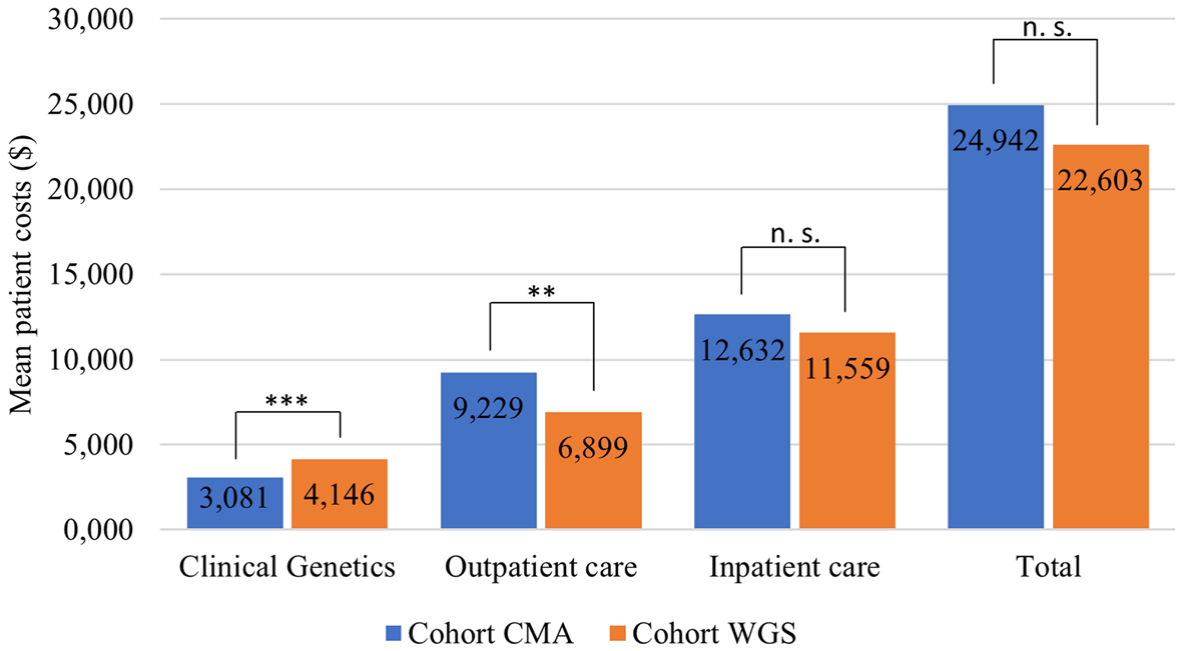
Mean patient costs ($) for each cohort calculated from referral for CMA/WGS testing and 2 years onwards (***P < 0.001; **P < 0.01; *P < 0.05; *n.s.* not significant).

The annual cost the year prior to referral year was similar in the WGS and CMA cohorts (P = 0.69). Cohort WGS had 9.4% lower mean total costs per patient compared to cohort CMA 2 years after the initial referral ($22,603 vs $24,942). Even though the mean cost of genetic tests was 34.5% ($1065) higher for cohort WGS, lower costs were observed for both outpatient and inpatient care, which were 25% ($230) and 8.5% ($1073) less (Fig. 2).

The complementary regression analysis (Supplemental Table S3) showed that costs were not affected by sex (P = 0.98) but younger patients were more costly (P < 0.001).

The difference in mean total cost per patient of $ − 2339 (Fig. 2) was not statistically significant ($ − 2339, 95% CI − 12,238–7561; P = 0.64). Among the sub-cost categories, the difference in mean outpatient care costs ($ − 2330, 95% CI − 3992 to (− 669); P = 0.006) and in genetic testing ($1065, 95% CI 834–1295; P < 0.001) were statistically significant. The mean cost difference in inpatient care was not statistically significant ($ − 1073, 95% CI − 10,704–8558; P = 0.83). The mean number of inpatient visits and hospitalization days were higher for cohort WGS (Table 2). Still, the mean cost for inpatient care was lower for cohort WGS. For outpatient care cohort WGS had both lower number of visits and lower costs. In the department of Clinical Genetics there a was a higher use of WGS tests and a lower use of CMA, Fragile X and exome tests for cohort WGS compared to cohort CMA. Cohort WGS also showed lower use of complimentary tests (grouped as “Other tests” in Table 2) as well as fewer consultations. For an overview of the unit prices of common genetic tests performed please see Supplemental Table S1.

**Table 2:** Mean patient health care consumption, calculated from referral and 2 years onwards.

	Cohort CMA	Cohort WGS	P-value (diff.)
Inpatient care			
Visits	0.983	1.326	0.39
Days hospitalized	7.842	13.438	0.24
Outpatient care			
Visits	57.778	53.202	0.36
Clinical genetics			
CMA	1.005	0.000	< 0.001
Fragile X	0.368	0.000	< 0.001
WGS	0.007	1.000	< 0.001
ES	0.134	0.000	< 0.001
Chromosomal analysis	0.029	0.045	0.49
MPS-verification	0.050	0.010	< 0.001
Other tests	0.272	0.224	0.52
Isolation of DNA	0.911	0.933	0.49
Cell culture	0.100	0.079	0.50
Consultations	0.524	0.281	< 0.001

### Long term diagnostic cost-trajectory for cohort CMA

Since cohort CMA consisted of referrals from 2015 (compared to cohort WGS from 2017) there was more data available to study the development of performed genetic tests in cohort CMA, beginning at the end of the 2-year period and ending at the end date of the data set from Clinical Genetics (2020-06-30). During this additional period the largest increases in tests per individual were changes in WGS testing (0.007 to 0.081), MPS verification tests (0.060 to 0.103) and consultations (0.407 to 0.519). Mean genetic test costs per individual in cohort CMA increased by 17% during this period (from $3081 as shown in Fig. 2 to $3620).

### Progressive diagnostic yield

During the 2-year period post referral Cohort WGS generated a diagnostic yield 23% higher than cohort CMA (24.7% for cohort WGS and 20.1% for cohort CMA). The difference was not statistically significant (0.046, 95% CI − 0.053 to 0.145; P = 0.36). The individuals who were diagnosed within 2 years from referral were younger (P = 0.003) at time of referral, and displayed a smaller share of NDD and higher share of affected newborn as main reason for referral (Supplementals Table S2).

**Figure 3:**
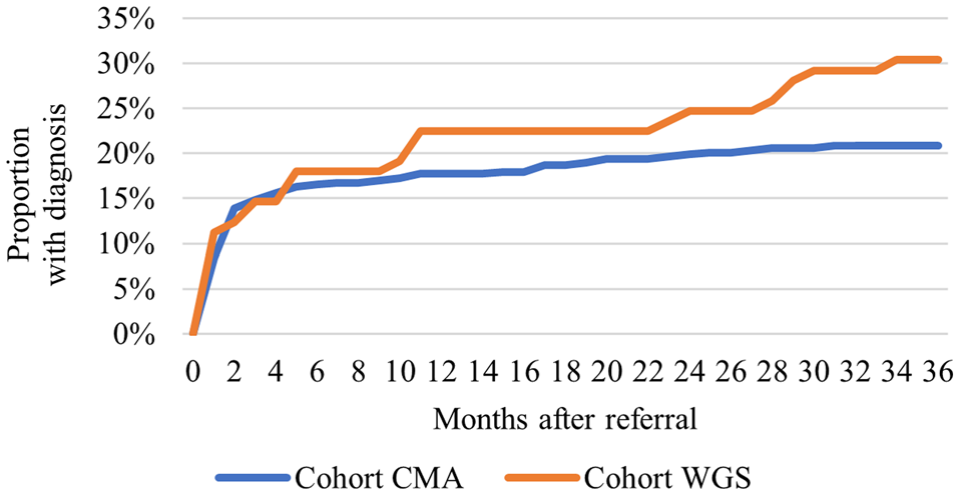
Cumulative progression of diagnostic yield per cohort.

Adding an additional year of follow up increased the diagnostic yield difference to 46% between the two cohorts (30.3% for cohort WGS and 20.8% for cohort CMA) (0.095, 95% CI − 0.009 to 0.200; P = 0.074). The cumulative progression of diagnostic yield is shown in Fig. 3.

### Cost effectiveness

As the mean costs for the WGS cohort were lower (n.s) and the diagnostic yield was higher (n.s) the use of WGS as a first-line test was deemed dominant albeit not statistically significant.

### Sensitivity analysis

In the sensitivity analysis Cohort WGS showed a significantly lower cost of genetic testing than Cohort CMA when the WGS verification test costs were set to zero and WGS testing costs to 50% ($ − 698, 95% CI − 906 to (− 489); P < 0.001). The total costs difference between the two cohorts was still not significant ($ − 4101, 95% CI − 14,002–5800; P = 0.42).

The costs for genetic testing were equal between the cohorts when WGS testing costs were set to 70% (given that the WGS verification test costs were set to zero).

## Discussion

The results from the study show that the costs when using WGS as a first-line diagnostic test were $2339 lower compared to the standard of care strategy during the first 2 years from referral. The diagnostic yield was 23% higher for cohort WGS during the same time period compared to the CMA group. Thus, from a cost-effectiveness perspective, the WGS test is dominant.

These main results should be interpreted with some caution as they are associated with statistical uncertainty, but may still be of importance when considering the allocation of scarce health care resources. The role of statistical significance in the prioritization of management strategies is unclear. Provided that decision makers must decide on using either WGS or CMA as a first-line of testing the numerical differences in mean costs and diagnostic yield indicate the expected outcome of using WGS rather than CMA will be positive, albeit with some uncertainty^27^. Prioritizing CMA instead of WGS would instead mean that expected outcomes will be negative, albeit also uncertain. Although the relevance of uncertainty for decision making may be questioned, it should be pointed out that the uncertainty around the estimated costs and diagnostic yield is a clear signal that further research in the field is needed.

The results of this study showed that while the difference in mean outpatient and clinical genetic cost was statistically significant the difference in mean inpatient cost was not. The reason for this statistical insignificant difference may be that this type of cost item occurs less frequently than, for example, outpatient care costs, and shows greater variation in cost size than the other categories. Inpatient care costs include, for example, surgery costs which can be both very high and rarely occurring.

The mean cost for genetic testing was significantly higher in cohort WGS but this difference may diminish over time. The lower diagnostic yield in cohort CMA will result in additional genetic testing, as confirmed by the observed cost development (+ 17%) in cohort CMA after the initial 2 years from referral. Since WGS is a more comprehensive test we hypothesize that cohort WGS, in comparison, would require less additional tests during an equivalent follow up period, decreasing the difference in mean genetic costs between the two cohorts.

Comparing costs of genetic tests between treatment centers is difficult since the price calculation models may differ. We used the 2020 pricelists at our unit as basis for the calculations presented in this study. Based on these, the cost of WGS and CMA was estimated to be $3434.6 and $1485.7, respectively. Included in the price for clinical WGS is sequencing reagents and service of instruments, bioinformatics processing and secure storage of the bigdata files as well as clinical interpretation and reporting of findings. It is also difficult to predict the future price development for WGS testing. Even if the reagent costs are lowered the increasing demand for bioinformatics development as well as training of highly specialized clinical geneticists will continue to keep the costs high for the foreseeable future.

The higher diagnostic yield for cohort WGS means that more individuals receive an understanding of his or her condition and an end to a diagnostic odyssey which can be costly, time consuming and psychologically burdensome to the patient and next of kin^13^. The increased knowledge can also be used to tailor treatment, identify additional conditions that are known to be associated with the diagnosis, as well as provide access to carrier testing and family planning for family members^13^.

The results from previous studies concerning the use of WGS were mixed. In a review article^20^ from 2018 the researchers did not find enough support to be able to draw a conclusion about its cost-effectiveness. The reviewed articles, among other things, lacked sufficient scope and were quickly outdated by the rapid pace of technological development. The authors instead concluded that the need for further research was great. Furthermore, in a hypothetical scenario analysis comparing WGS and CMA testing for autism spectrum disorders, Tsiplova et al.^28^ found WGS to be more expensive (adding 2108–4776CAD per year) with an ICER of 26,020–58,959CAD depending on type of WGS-test. This estimate only included costs for genetic testing and was not based on registry data, we believe that the more comprehensive results from our study contribute to this still relatively unexplored area of research.

The results from the current study are based on assumptions about comparability between the cohorts and the validity of the VAL databases cost items. Additionally, the interpretation of changes in actual health effects is limited due to the use of diagnostic yield as measurement. A health economic measurement of quality adjusted life-years would be more informative, measuring i.e. the gain in quality of life that is associated with the increased knowledge of the condition, when diagnosed, by both the individual and next of kin. However, this was beyond the scope of the study and there is, to our knowledge, little knowledge or ongoing research investigating these effects.

Regarding future research, the uncertainty that accompanies the results could be reduced by increasing the studied time interval or the number of individuals examined in future studies. It is also beneficial to re-evaluate whole genome sequencing as a method in the future since both the costs of testing and the potential to find additional diagnoses are likely to change over time. We believe that costs for WGS testing will decrease and that this, as shown in our sensitivity analysis, will accentuate the dominance of WGS as a first line genetic test. Additional aspects of future interest would be to measure the effects of diagnosis in quality adjusted life-years (QALYs), as well as expanding the scope of the study to include a wider, societal, perspective.

## Conclusion

WGS as a first-line diagnostic test for individuals with neurodevelopmental disorders is associated with statistically non-significant lower costs and higher diagnostic yield compared with CMA. WGS is therefore considered a dominant strategy. This indicates that prioritizing WGS over CMA in health care decision making will yield positive expected outcome, as well as showing a need for more extensive research in order to reduce the uncertainty in the estimations.

## Supplementary Information

Supplementary Tables.

## Data Availability

The datasets generated and/or analyzed during the current study are not publicly available, since the participants of the study have not agreed to share their data publicly, but are available from the corresponding author on reasonable request.

## Abbreviations

CNV: Copy number variant
CMA: Chromosomal microarray
ID: Intellectual disability
INDEL: Insertions and deletions
IQ: Intelligence quotient
MPS: Massive parallel sequencing
NDD: Neurodevelopmental disorders
QALY: Quality adjusted life year
SNV: Single nucleotide variant
STR: Short tandem repeat expansion
SV: Structural variant
WGS: Whole genome sequencing
