# Supplementary material for: The cost-effectiveness of whole genome sequencing in neurodevelopmental disorders
Source: Sci Rep. 2023 Apr 27;13:6904. doi: 10.1038/s41598-023-33787-8 (PMC10140052; doi:10.1038/s41598-023-33787-8)
Supplement: Supplementary file 1 — Supplementary Tables. [file 41598_2023_33787_MOESM1_ESM.docx]

Table S1. Unit costs for genetic tests performed (includes analysis and reporting of results)

| **Genetic test** | **Unit price 2020 (USD)** |
| --- | --- |
| CMA | 1486 |
| Fragile X | 771 |
| WGS | 3435 |
| ES | 3139 |
| Chromosomal analysis | 631 |
| Isolation of DNA | 342 |
| Cell culture | 626 |

Table S2. Characteristics of diagnosed and undiagnosed individuals

|  | **Diagnosed^d^** | **Undiagnosed^d^** | **P-value. Diff.** |
| --- | --- | --- | --- |
| Sex (female) | 42% (n=44) | 32% (n=129) | 0.083 |
| Age^a^ (mean years; min/max/median) | 4;0.0/14.4/2.8 | 5.3;0.0/15.5/4.3 | 0.003 |
|  |  |  |  |
| Main reason for referral^b^ |  |  |  |
| Neurodevelopmental disorder | 70% (n=74) | 85% (n=340) | 0.002 |
| Affected newborn (<3months) | 18% (n=19) | 8% (n=33) | 0.016 |
| Other^c^ | 15% (n=16) | 9% (n=37) | 0.123 |

^a^ At time of referral

^b^ Twelve newborns showed developmental delay that was obvious as early as under three months of age, hence twelve patients are included in both Neurodevelopmental disorder group and Affected newborn (<3 months) group, hence the percentages will not add up.

^b^ Epilepsy, congenital malformations or suspected neuromuscular disease

^c^ The Diagnosed group contains all diagnosed individuals from Cohort CMA and Cohort WGS combined, after two years from referral. The Undiagnosed group contains all undiagnosed individuals from Cohort CMA and Cohort WGS combined, after two years from referral.

Table S3. Regression analysis, effects of age and sex on total costs

| *Regression Statistics* | |
| --- | --- |
| Multiple R | 0.2694 |
| R Square | 0.0726 |
| Adjusted R Square | 0.0671 |
| Standard Error | 558192 |
| Observations | 507 |

|  | *Coefficients* | *Standard Error* | *t Stat* | *P-value* | *Lower 95%* | *Upper 95%* |
| --- | --- | --- | --- | --- | --- | --- |
| Intercept | 421 250 | 44 666 | 9.431 | <0.001 | 333 495 | 509 005 |
| Kohort WGS | -32 431 | 65 213 | -0.497 | 0.619 | -160 556 | 95 693 |
| Ålder vid remiss | -37 862 | 6 043 | -6.265 | <0.001 | -49 735 | -25 990 |
| Kön (M=0) | -1 411 | 52 311 | -0.027 | 0.978 | -104 186 | 101 365 |
